# Integrated physiological and multi-omics analyses identify a *SmWRKY4*–*SmAPX2* regulatory module associated with cold adaptation in *Sapindus mukorossi*

**DOI:** 10.3389/fpls.2026.1841628

**Published:** 2026-06-09

**Authors:** Anguo Qi, Huijuan Chen, Zhaokun Zhi, Huina Wang, Xinfang Yan

**Affiliations:** 1 School of Horticulture and Landscape Architecture, Henan Institute of Science and Technology, Xinxiang, Henan, China; 2 The Forestry Workstation of Xinxiang City, Xinxiang, Henan, China; 3 The Forest Bureau of Xinxiang City, Xinxiang, Henan, China

**Keywords:** ascorbate–glutathione cycle, cold adaptation, reactive oxygen species, *Sapindus mukorossi*, *SmAPX2*, *SmWRKY4*

## Abstract

The northward cultivation of *Sapindus mukorossi* Gaertn. has long been constrained by winter low temperatures, making it important to clarify the mechanisms underlying cold adaptation for germplasm utilization and molecular improvement. In this study, previously screened cold-tolerant (CT) and cold-sensitive (CS) material groups were used to examine the basis of cold adaptation through phenotypic observation, anatomical analysis, physiological and biochemical measurements, transcriptomic and metabolomic profiling, weighted gene co-expression network analysis (WGCNA), functional validation, and transcriptional regulatory assays. Compared with CS, CT maintained higher branch structural stability, showed less cellular damage, and exhibited a stronger capacity to sustain reactive oxygen species (ROS) scavenging and redox homeostasis under low-temperature stress. Integrated multi-omics analyses further showed that these differences were associated with coordinated reprogramming of sugar and amino acid metabolism, together with changes in ascorbate–glutathione (AsA–GSH)-related processes. On this basis, *SmAPX2* was selected as a key candidate gene involved in antioxidant-mediated cold tolerance. Functional analyses showed that *SmAPX2* increased ascorbate peroxidase (APX) activity and reduced the accumulation of H_2_O_2_, malondialdehyde (MDA), relative electrolyte conductivity (REC), and O_2_·⁻ under low-temperature conditions, indicating that it contributes to cold tolerance by enhancing ROS detoxification and alleviating membrane damage. Further analyses showed that *SmWRKY4* bound to the *SmAPX2* promoter and enhanced its transcriptional activity, with the proximal W-box contributing substantially to *SmWRKY4*-mediated promoter activation. These findings suggest that the *SmWRKY4*–*SmAPX2* regulatory module may participate in cold adaptation-associated antioxidant responses in *S. mukorossi*.

## 1 Introduction

*Sapindus mukorossi* Gaertn. is a woody tree species of considerable economic, ecological, and horticultural value (Liu et al., 2022; Sahito et al., 2023). The fruit pericarp contains abundant saponins, which confer natural surfactant properties and support its use in detergent and related applications (Sochacki and Vogt, 2022), while the seed kernels are characterized by a relatively high oil content and have been explored as a potential resource for biodiesel production (Zhou et al., 2024). As one of the most extensively utilized species in the genus *Sapindus*, *S. mukorossi* has a solid resource base and considerable industrial potential (Liu et al., 2022). However, its regional cultivation remains markedly constrained by environmental adaptability, particularly by overwintering safety under low-temperature conditions, which has become a major factor limiting its northward introduction and efficient utilization (Liu et al., 2021). Therefore, screening cold-tolerant germplasm and clarifying the mechanisms underlying cold adaptation are important not only for improving the application potential of *S. mukorossi* in colder regions, but also for its regional cultivation and molecular improvement.

Low temperature is a major abiotic stress that restricts plant growth, geographical distribution, and productivity (Landi et al., 2021). In woody plants, low-temperature injury is manifested not only as damage to shoots and buds, but also as disruption of tissue structure, impairment of membrane systems, and imbalance of cellular physiology and metabolism. Increased membrane permeability and aggravated membrane lipid peroxidation are usually early hallmarks of cold injury, whereas excessive accumulation of reactive oxygen species (ROS) under low temperature can further amplify oxidative damage and disrupt cellular homeostasis (Wang et al., 2019). To alleviate cold-induced injury, plants generally maintain cellular homeostasis by accumulating osmotic adjustment substances and activating antioxidant defense systems. Accordingly, relative electrolyte conductivity (REC), malondialdehyde (MDA), proline (Pro), soluble sugars (SS), soluble protein (SP), and the activities of superoxide dismutase (SOD), peroxidase (POD), catalase (CAT), and ascorbate peroxidase (APX) have been widely used to evaluate plant cold tolerance and low-temperature responses (Landi et al., 2021). Among these processes, disruption of ROS metabolism is considered an important physiological basis of stress-induced injury, whereas APX, as one of the key enzymes responsible for H_2_O_2_ scavenging in the ascorbate–glutathione cycle, plays a crucial role in maintaining cellular redox homeostasis and enhancing stress tolerance (Yao et al., 2021).

Although phenotypic observations and physiological and biochemical indices can effectively characterize differences in cold tolerance among materials, they are insufficient to fully explain the molecular basis underlying cold adaptation. In recent years, transcriptomics and metabolomics have become important approaches for dissecting plant stress adaptation mechanisms (Li et al., 2023; Song et al., 2023; Xiao et al., 2024). Transcriptomic analysis can identify differentially expressed genes and their regulatory pathways at the whole-genome level, whereas metabolomic analysis can reveal differentially accumulated metabolites and the characteristics of cold-induced metabolic reprogramming. When integrated with co-expression network analysis, these approaches help establish systematic links among changes in gene expression, metabolic regulation, and the formation of cold-tolerant phenotypes, thereby improving the accuracy of key pathway identification and core candidate gene screening (Ning et al., 2024; Wang et al., 2025a). Previous studies have shown that plant cold adaptation is commonly accompanied by substantial reprogramming of sugar metabolism, amino acid metabolism, phenylpropanoid metabolism, plant hormone signal transduction, mitogen-activated protein kinase (MAPK) signaling, and antioxidant-related processes (Song et al., 2023; Li et al., 2024; Sun et al., 2024). Therefore, integrating multi-omics analyses to identify key cold-tolerance factors from complex response networks represents an effective strategy for deepening our understanding of the molecular mechanisms underlying cold adaptation.

Among the pathways involved in low-temperature responses, the ascorbate–glutathione cycle represents a major ROS-scavenging system and is closely linked to upstream transcriptional regulation. In this cycle, ascorbate peroxidase (APX) catalyzes the reduction of H_2_O_2_ and contributes to the maintenance of cellular redox balance (Foyer and Kunert, 2024). Increasing evidence indicates that APX activity is associated with enhanced tolerance to abiotic stress, partly through limiting the accumulation of H_2_O_2_ and lipid peroxidation products such as MDA. APX2 has been reported to play a role in modulating oxidative damage under stress conditions, suggesting its involvement in plant adaptation to adverse environments (Zeng et al., 2022; Li, 2023; Yoshimura and Ishikawa, 2024). Meanwhile, the WRKY transcription factor family is widely involved in plant stress responses and can regulate downstream stress-responsive genes by recognizing W-box elements in promoters, thereby playing important roles in temperature stress signal transduction (Rushton et al., 2010). In addition, WRKY family members can act cooperatively with other transcription factors or regulatory proteins to form more complex stress regulatory networks (Jiang et al., 2017). Therefore, further elucidation of the upstream regulatory relationships between WRKY transcription factors and APX or other antioxidant-related genes within the ROS-scavenging system is important for clarifying the molecular basis underlying *S. mukorossi* cold adaptation.

At present, research on cold adaptation in *S. mukorossi* remains largely limited to phenotypic description and physiological characterization, and systematic investigation integrating transcriptomics, metabolomics, and co-expression network analysis is still lacking. In particular, the roles of APX-related functional genes and their potential regulation by WRKY transcription factors in *S. mukorossi* cold adaptation remain poorly understood. Based on this background, the present study used previously screened cold-tolerant (CT) and cold-sensitive (CS) materials to investigate the physiological and molecular basis of cold adaptation. Differences in phenotypic traits, anatomical structure, and physiological and biochemical responses between the two types of materials under low-temperature stress were first compared. Subsequently, integrated transcriptomic and metabolomic analyses were performed at key time points, and weighted gene co-expression network analysis (WGCNA) was applied to identify key modules and candidate genes associated with cold adaptation. On this basis, the key candidate gene *SmAPX2* was selected for functional validation, and the transcriptional regulation of *SmAPX2* by the upstream transcription factor *SmWRKY4* was further characterized. This study aimed to clarify the physiological and molecular processes associated with cold adaptation in *S. mukorossi* from multiple levels, including physiological response, metabolic reprogramming, gene function, and transcriptional regulation, and to provide a theoretical basis for cold-tolerant germplasm evaluation, identification of key genes, and future molecular improvement of this species.

## 2 Materials and methods

### 2.1 Plant materials and low-temperature treatment

In our previous study, freezing injury was evaluated in 126 *Sapindus mukorossi* materials under natural extreme low-temperature conditions, and their cold tolerance was comprehensively assessed. Based on the comprehensive ranking results, 10 cold-tolerant (CT) and 10 cold-sensitive (CS) materials were selected. For each selected material, three individual plants were included, resulting in 30 CT and 30 CS individuals. The provenance, material ID, plant number, and field plot coordinate of each selected individual are provided in **Supplementary Table 1**. For field phenotypic observation and anatomical analysis, three representative accessions from each cold-tolerance type were selected from these screened materials.

For artificial low-temperature treatment, five healthy one-year-old branches were collected from each individual plant. Thus, 150 branches were obtained for each cold- response type and randomly divided into 30 groups, with five branches per group. Each group was pooled as one biological sample. A room-temperature control at 25 °C was set as CK, and low-temperature treatments were conducted at −14 °C for 2, 4, 8, 12, 24, 48, and 72 h. At each time point, three biological replicates were sampled. After treatment, tissues from the middle portion of the branches were collected without removing the bark. Samples were either used immediately for physiological and biochemical measurements or frozen in liquid nitrogen and stored at −80 °C for subsequent transcriptomic and metabolomic analyses.

All source plants were four-year-old seedling-derived individuals. Therefore, the CT and CS groups should be regarded as representative contrasting cold- response groups selected from a broader field evaluation, rather than genetically identical clonal lines.

### 2.2 Field phenotypic observation and anatomical analysis of branch structure

Freezing injury phenotypes of one-year-old branches from CT and CS materials were observed and photographed under field conditions after natural overwintering, following general criteria for woody plant frost injury assessment (Neuner, 2014). To quantify differences in spring bud break, bud break was evaluated in March 2024 before subsequent sampling. For the four-year-old seedlings, the apical 20-cm segment of the one-year-old branch was used as a standardized region for bud counting, and three individual plants were evaluated for each selected material. The total number of visible bud positions and sprouted buds within this segment was recorded. Sprouted buds were defined as buds showing visible bud break or new shoot emergence, and non-sprouted bud positions were defined as visible bud positions without bud break. Bud break rate and non-sprouting rate were calculated relative to the total number of visible bud positions.

Branch anatomical structure was analyzed using the paraffin section method (Alshammari et al., 2024). After natural low-temperature exposure, tissues from the middle portion of one-year-old branches were collected from representative plants, fixed, processed through standard paraffin embedding procedures, sectioned at 12–14 μm, double-stained with safranin O and fast green, and observed under an upright light microscope (Eclipse E100, Nikon, Tokyo, Japan). Images were captured using a microscopic imaging system (DS-U3, Nikon, Tokyo, Japan). For quantitative anatomical analysis, six independent cross-sections were analyzed for each cold-response type. Branch radius, cortex thickness, phloem thickness, and xylem thickness were measured using CaseViewer 3.0 and ImageJ 1.54. Cortex, phloem, and xylem rates were expressed as percentages of branch radius, and the xylem/phloem ratio was determined from the corresponding thickness values.

### 2.3 Determination of physiological and biochemical indices

REC was measured using the electrolyte leakage method (Prášil and Zámečnıík, 1998). Fresh branch tissues were cut into small pieces and immersed in deionized water. After shaking at room temperature, the initial conductivity was recorded. The samples were then boiled in a water bath, cooled to room temperature, and the final conductivity was measured. REC was calculated from the initial and final conductivity values. MDA content and the activities of SOD, POD, CAT, and APX were determined using assay kits from Beijing Solarbio Science & Technology Co., Ltd. (Beijing, China), with catalog numbers BC0025, BC0175, BC0095, BC0205, and BC0225, respectively. Pro, SS, and SP were measured using assay kits from Suzhou Grace Biotechnology Co., Ltd. (Suzhou, China), with catalog numbers G0111W48, G0501W48, and G0418W, respectively. All assays were performed according to the manufacturers’ instructions, as described in previous plant stress physiology studies (Gu et al., 2021; Zhang et al., 2022), with three biological replicates for each treatment. Absorbance was measured using a multifunctional microplate reader (Synergy H1, BioTek, Winooski, VT, USA) or a UV–visible spectrophotometer (UV-2600, Shimadzu, Kyoto, Japan).

### 2.4 Transcriptomic and metabolomic sequencing and data analysis

#### 2.4.1 Sample selection and sequencing

Samples collected at CK, 12, 24, 48, and 72 h were selected for transcriptomic and metabolomic analyses. The 2, 4, and 8 h time points were mainly used to characterize the early physiological response dynamics to low-temperature stress, whereas the omics analyses were designed to focus on representative stages at which the divergence between CT and CS became clearer and more sustained. Based on the physiological results, relatively stable differences between CT and CS in membrane injury, osmotic adjustment, and antioxidant-related indices were more evident from 12 h onward. Therefore, CK, 12, 24, 48, and 72 h were selected to represent the control, early established response stage, intermediate stage, and prolonged stress stages for subsequent integrated transcriptomic and metabolomic analyses. Three independent biological replicates were included for each treatment. After sampling, all materials were immediately frozen in liquid nitrogen and stored at −80 °C.

#### 2.4.2 Transcriptomic data analysis

Total RNA concentration and purity were measured using a NanoDrop 2000 spectrophotometer (Thermo Fisher Scientific, Waltham, MA, USA). After quality assessment, RNA-seq libraries were constructed and sequenced. Raw reads were processed by removing adaptor sequences, low-quality reads, and reads containing excessive unknown bases to obtain clean reads. Clean reads were aligned to the *S. mukorossi* reference genome and corresponding annotation files obtained from SapBase (Sapindaceae Genome Database; Xue et al., 2022; accessed on 18 March 2025). The *S. mukorossi* genome resource used in this study includes genome FASTA and GFF3 annotation files, with 14 chromosomes, a genome size of 432 Mb, and 30,184 annotated genes. Gene expression levels were quantified and normalized as fragments per kilobase of transcript per million mapped reads (FPKM). Differentially expressed genes (DEGs) were identified using |log_2_FoldChange| ≥ 1 and a false discovery rate (FDR) < 0.05 as the screening thresholds (Lu et al., 2022). Principal component analysis (PCA), Pearson’s correlation analysis, hierarchical clustering analysis, and Kyoto Encyclopedia of Genes and Genomes (KEGG) pathway enrichment analysis were then performed. The raw transcriptome data have been deposited in the NCBI Sequence Read Archive (SRA) under BioProject accession number PRJNA1438190.

#### 2.4.3 Metabolomic data analysis

After quality-control filtering, metabolites were annotated based on the in-house database and public databases. Differentially accumulated metabolites (DAMs) were identified using variable importance in projection (VIP) ≥ 1 and |log_2_FoldChange| ≥ 1 as the screening thresholds (Lu et al., 2022; Li et al., 2023). PCA, Pearson’s correlation analysis, clustering analysis, and KEGG pathway enrichment analysis were subsequently performed.

#### 2.4.4 Integrated transcriptomic and metabolomic analysis and WGCNA

Integrated transcriptomic and metabolomic analysis was conducted by comparing the KEGG enrichment results of DEGs and DAMs to identify pathways jointly responsive to low-temperature stress. Weighted gene co-expression network analysis (WGCNA) was then performed based on all expressed genes (Langfelder and Horvath, 2008). After matching the gene expression matrix with physiological traits and key low temperature-related metabolites across common samples, different soft-thresholding powers (1–10 and 12–20, with an increment of 2) were tested using the WGCNA package. The optimal parameter was selected according to the scale-free topology fit index and mean connectivity. When the scale-free topology fit index exceeded 0.85, a power of 12 was chosen for network construction. The network was built using an unsigned topology, with the minimum module size set to 30 genes and the module merging threshold set to 0.25. Correlations between module eigengenes and physiological traits or key metabolites were further calculated to identify modules and candidate genes associated with cold adaptation.

### 2.5 Candidate gene screening and qRT-PCR validation

Twelve candidate genes were chosen for qRT-PCR validation according to WGCNA analysis, functional annotation, and enrichment of cold-related pathways. Total RNA was extracted using a plant RNA extraction kit (Vazyme, Nanjing, China). RNA quality was examined by 1% agarose gel electrophoresis, and its concentration and purity were measured with a NanoDrop 2000 spectrophotometer. First-strand cDNA was then synthesized using a reverse transcription kit (Vazyme, Nanjing, China). qRT-PCR was performed using SYBR qPCR Mix (Vazyme, Nanjing, China) on a CFX96 Real-Time PCR System (Bio-Rad, Hercules, CA, USA). The amplification program consisted of an initial denaturation at 95 °C for 30 s, followed by 40 cycles of 95 °C for 5 s and 60 °C for 30 s, and then melting curve analysis. *SmACTIN9* (whz_001081) was used as the internal reference gene (Xu et al., 2022), and relative expression levels were calculated using the 2^−ΔΔCt^ method (Livak and Schmittgen, 2001). Three biological replicates and three technical replicates were included for each sample. Primer sequences are listed in **Supplementary Table S2**.

### 2.6 Subcellular localization prediction of *SmAPX2*

The subcellular localization of *SmAPX2* was predicted using four online tools: WoLF PSORT, DeepLoc 2.1, Plant-mPLoc, and CELLO. Prediction results from different tools were compared to infer the putative cellular compartment of *SmAPX2*. The analyses were performed in April 2026.

### 2.7 Functional validation of *SmAPX2*

#### 2.7.1 Heterologous expression analysis in yeast

The coding sequence of *SmAPX2* was cloned into the yeast expression vector pESC-Trp, and the resulting construct, together with the empty vector control, was transformed into *Saccharomyces cerevisiae* strain BY4741 (Chen et al., 2023). Positive transformants were identified on selective medium and used for subsequent low-temperature tolerance analysis. For the spot assay, yeast cultures were grown to the logarithmic phase, adjusted to an OD_600_ of 0.8, and subjected to 10-fold serial dilutions. Aliquots of each dilution were spotted onto the corresponding selective medium and incubated at 30 °C or 17 °C, after which colony growth was recorded. For the liquid culture assay, recombinant yeast cells and empty-vector controls were inoculated into liquid medium and cultured with shaking at 17 °C. OD_600_ values were measured every 4 h to generate growth curves.

#### 2.7.2 Genetic transformation of *Arabidopsis thaliana* and low-temperature treatment

The coding sequence of *SmAPX2* was cloned into the plant overexpression vector pCAMBIA1302 and introduced into *Agrobacterium tumefaciens* GV3101. Wild-type *Arabidopsis thaliana* (Col-0) plants were transformed using the floral-dip method (Clough and Bent, 1998; Zhang et al., 2006). Putative transformants were first identified by resistance screening and then verified by molecular assays. Three independent homozygous lines with higher expression levels, designated OE-1, OE-2, and OE-3, were chosen for further analysis. Wild-type plants and overexpression lines were grown in an artificial climate chamber at 22 °C under a 16 h light/8 h dark photoperiod for 3 weeks and then transferred to 4 °C for 24 h. After treatment, plant phenotypes were documented, and *SmAPX2* expression, APX activity, H_2_O_2_ content, MDA content, REC, and O_2_·⁻ content were measured.

### 2.8 Verification of the regulatory relationship between *SmWRKY4* and *SmAPX2*

#### 2.8.1 Promoter cis-element analysis and expression correlation analysis

A 2000-bp promoter sequence upstream of the translation initiation site of *SmAPX2* was retrieved and analyzed for cis-acting elements using the PlantCARE database (Lescot et al., 2002). The predicted results are listed in **Supplementary Table S3**. Based on the distribution of W-box elements and the expression patterns of candidate transcription factors, *SmWRKY4* was selected as a potential upstream regulator. The expression correlation between *SmWRKY4* and *SmAPX2* was further analyzed based on the qRT-PCR data.

#### 2.8.2 Yeast one-hybrid assay

A fragment of the *SmAPX2* promoter was cloned into the pAbAi vector to construct the bait plasmid, and the coding sequence of *SmWRKY4* was cloned into the pGADT7 vector to construct the effector plasmid. The yeast one-hybrid (Y1H) assay was performed according to the instructions of the Matchmaker Gold Yeast One-Hybrid System (Takara/Clontech, Mountain View, CA, USA). After integration of the bait plasmid into the yeast genome, self-activation was tested under different concentrations of Aureobasidin A (AbA), and 400 ng·mL⁻¹ AbA was selected as the screening concentration. The effector plasmid was then transformed into the bait yeast strain, and yeast growth on SD/-Leu medium containing AbA was observed to determine whether *SmWRKY4* could bind to the tested *SmAPX2* promoter fragment.

#### 2.8.3 Dual-luciferase reporter assay

The *SmAPX2* promoter was cloned into the pGreenII 0800-LUC vector to construct the reporter plasmid, and the coding sequence of *SmWRKY4* was cloned into the pGreenII 62-SK vector to construct the effector plasmid. The recombinant plasmids were individually introduced into *Agrobacterium* GV3101 and then co-infiltrated into *Nicotiana benthamiana* leaves. The bacterial suspension was adjusted to an OD_600_ of 0.8. After incubation in darkness for 12 h, the infiltrated plants were returned to normal growth conditions. Luminescence signals were detected 48 h after infiltration, and the LUC/REN ratio was measured using a Dual-Luciferase Reporter Assay System (Promega, Madison, WI, USA). Three independent biological replicates were included for each treatment.

To further examine the contribution of the predicted W-box elements to *SmWRKY4*-mediated activation, site-directed mutations were introduced into the *SmAPX2* promoter. Two W-box motifs located at approximately −260 bp and −1639 bp upstream of the translation start site were mutated individually or simultaneously. The core W-box sequence TGAC was mutated to TAAC. The mutant promoter fragments, designated ProSmAPX2^m260^, ProSmAPX2^m1639^, and ProSmAPX2^m260+m1639^, were cloned into the pGreenII 0800-LUC reporter vector. These reporter constructs were co-infiltrated with the *SmWRKY4* effector construct into *Nicotiana benthamiana* leaves as described above. Relative promoter activity was determined based on the LUC/REN ratio.

### 2.9 Data processing and statistical analysis

Raw data were organized using Microsoft Excel 2019. Statistical analyses and figure preparation were performed using SPSS 26.0 (IBM Corp., Armonk, NY, USA), GraphPad Prism 9.0 (GraphPad Software, San Diego, CA, USA), and R 4.3.2 (R Foundation for Statistical Computing, Vienna, Austria). Data are presented as mean ± SD unless otherwise stated.

Bud break rate and quantitative anatomical traits between CT and CS were analyzed using Welch’s t-test. Physiological and biochemical indices and qRT-PCR data were compared between CT and CS at each time point using Student’s t-test. Student’s t-test was also used for two-group comparisons in functional validation and dual-luciferase assays. For comparisons among more than two groups, one-way analysis of variance (ANOVA) followed by Duncan’s multiple range test was performed. Differences were considered statistically significant at *P* < 0.05.

Principal component analysis (PCA), Pearson’s correlation analysis, heatmap visualization, and co-expression network-related analyses were performed in R. Final figure assembly was completed using Adobe Illustrator 2023.

## 3 Results

### 3.1 Phenotypic and anatomical differences between CT and CS under natural low-temperature conditions

**Figure 1:**
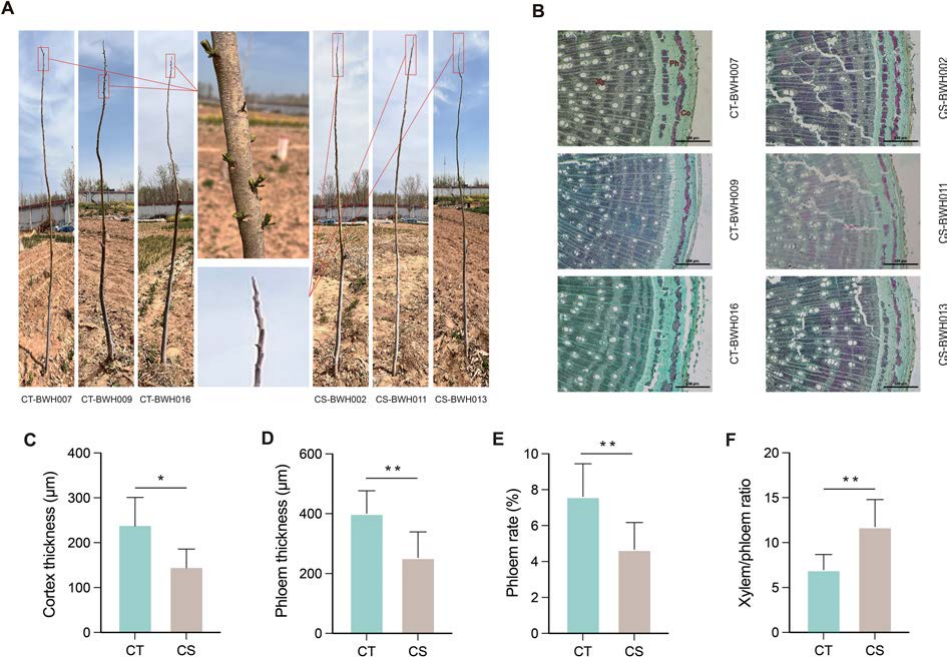
Phenotypic and anatomical differences between CT and CS under natural low-temperature conditions. **(A)** Branch phenotypes of representative cold-tolerant (CT; CT-BWH007, CT-BWH009, and CT-BWH016) and cold-sensitive (CS; CS-BWH002, CS-BWH011, and CS-BWH013) *Sapindus mukorossi* materials after natural low-temperature exposure. **(B)** Paraffin section-based cross-sectional anatomy of one-year-old branches from representative CT and CS materials. Co, cortex; Ph, phloem; Xy, xylem. **(C)** Cortex thickness. **(D)** Phloem thickness. **(E)** Phloem rate. **(F)** Xylem/phloem ratio. Data are presented as mean ± SD based on six independent sections per group. Asterisks indicate significant differences between CT and CS according to Welch’s t-test (*P < 0.05, **P < 0.01).

To verify the reliability of the preliminary screening results obtained under natural extreme low-temperature conditions, one-year-old branches from representative cold-tolerant (CT) and cold-sensitive (CS) materials were examined by field phenotypic observation and paraffin section-based cross-sectional analysis (**Figure 1**). The CT materials showed relatively fuller branches and partial bud sprouting, whereas the CS materials showed more slender branches, lower bud fullness, and more evident low-temperature injury symptoms (**Figure 1A**). To further quantify the field phenotypic differences after natural overwintering, bud break was evaluated within the apical 20-cm segment of the one-year-old branch. CT materials showed visible bud break within this segment, with an average bud break rate of 70.90%, whereas no sprouted buds were observed in the corresponding segment of CS materials. The bud break rate was significantly higher in CT than in CS (**Supplementary Table S4-1**).

Cross-sectional observations further revealed clearer tissue stratification and better organization of the cortex, phloem, and xylem in CT materials, while CS materials displayed more pronounced tissue loosening, local collapse, phloem disorganization, and blurred tissue boundaries (**Figure 1B**). Quantitative anatomical analysis supported these microscopic observations. Branch radius, xylem thickness, and xylem rate did not differ significantly between CT and CS. In contrast, CT showed significantly greater cortex thickness, phloem thickness, cortex rate, and phloem rate, whereas the xylem/phloem ratio was significantly higher in CS, mainly reflecting the reduced phloem thickness and proportion in CS materials (**Figures 1C–F**). These results indicate that CT maintained better cortical and phloem integrity after natural low-temperature exposure, whereas CS exhibited greater disruption of these tissues. The raw data and statistical results are provided in **Supplementary Table S4-2**.

### 3.2 Differences in physiological and biochemical responses between CT and CS under low-temperature stress

**Figure 2:**
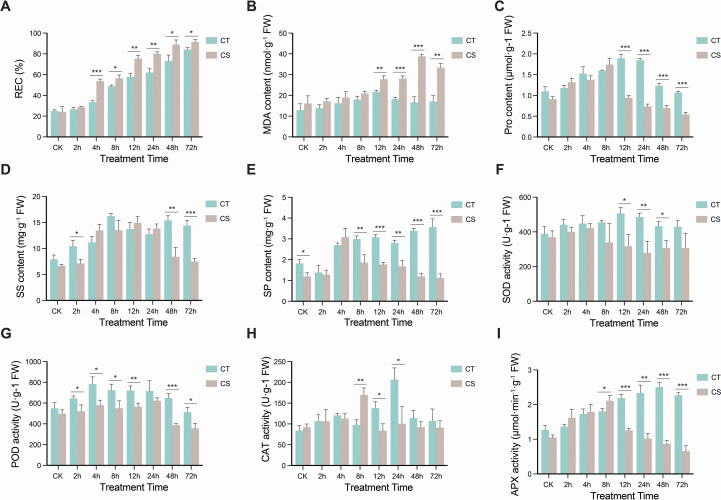
Physiological and biochemical responses of CT and CS under low-temperature stress. **(A)** Relative electrolyte conductivity (REC). **(B)** Malondialdehyde (MDA) content. **(C)** Proline (Pro) content. **(D)** Soluble sugar (SS) content. **(E)** Soluble protein (SP) content. **(F)** Superoxide dismutase (SOD) activity. **(G)** Peroxidase (POD) activity. **(H)** Catalase (CAT) activity. **(I)** Ascorbate peroxidase (APX) activity. One-year-old branches were treated at −14 °C for CK (0 h), 2 h, 4 h, 8 h, 12 h, 24 h, 48 h, and 72 h. Data are presented as mean ± SD (n = 3). Asterisks indicate significant differences between CT and CS at the same time point according to Student’s t-test (*P < 0.05, **P < 0.01, ***P < 0.001).

To compare the physiological responses of CT and CS under prolonged low-temperature stress, membrane injury-related indices, osmotic adjustment substances, and antioxidant enzyme activities were determined in one-year-old branches treated at −14 °C for different durations (**Figure 2**). The raw data used for statistical analysis are provided in **Supplementary Table S5**. REC increased progressively with treatment duration in both CT and CS, indicating a gradual increase in membrane permeability under low-temperature stress (**Figure 2A**). However, REC was generally higher in CS than in CT from 4 h onward, with significant differences observed at 4, 8, 12, 24, 48, and 72 h, suggesting that membrane injury occurred earlier and was more severe in CS. MDA content also showed an overall increasing trend (**Figure 2B**). Although the difference between the two materials was relatively small at the early stage, MDA became significantly higher in CS from 12 h onward and remained so until 72 h, indicating more pronounced membrane lipid peroxidation in CS under sustained low-temperature stress.

Low-temperature treatment induced the accumulation of osmotic adjustment substances in both CT and CS, with CT maintaining higher levels at most time points (**Figures 2C–E**). Pro content increased during the early stage, peaked at 12–24 h, and then declined, but remained higher in CT than in CS from 24 to 72 h (**Figure 2C**). SS content varied over time and was higher in CT at 2, 48, and 72 h (**Figure 2D**). SP levels in CT remained relatively stable during most of the treatment period and exceeded those in CS at several time points, particularly at 8, 12, 24, 48, and 72 h (**Figure 2E**). Overall, CT maintained higher levels of osmotic adjustment-related substances under prolonged low-temperature conditions, indicating stronger osmotic adjustment capacity than CS. Changes in antioxidant enzyme activities highlighted differences between CT and CS under low-temperature stress (**Figures 2F–I**). SOD activity remained higher in CT during the middle and late stages, with differences observed at 12, 24, and 48 h (**Figure 2F**). POD activity increased earlier in CT and stayed above that of CS at 4, 8, 12, 48, and 72 h (**Figure 2G**). CAT activity was transiently higher in CS at 8 h, but became significantly higher in CT at 12 and 24 h (**Figure 2H**). APX activity increased markedly in both materials at the early stage, but from 12 h onward it remained significantly higher in CT through 72 h (**Figure 2I**). Overall, these results indicate that CT experienced less membrane damage and maintained stronger osmotic adjustment and antioxidant enzyme activities than CS under prolonged low-temperature stress.

### 3.3 Transcriptomic and metabolomic divergence between CT and CS in response to low-temperature stress

**Figure 3:**
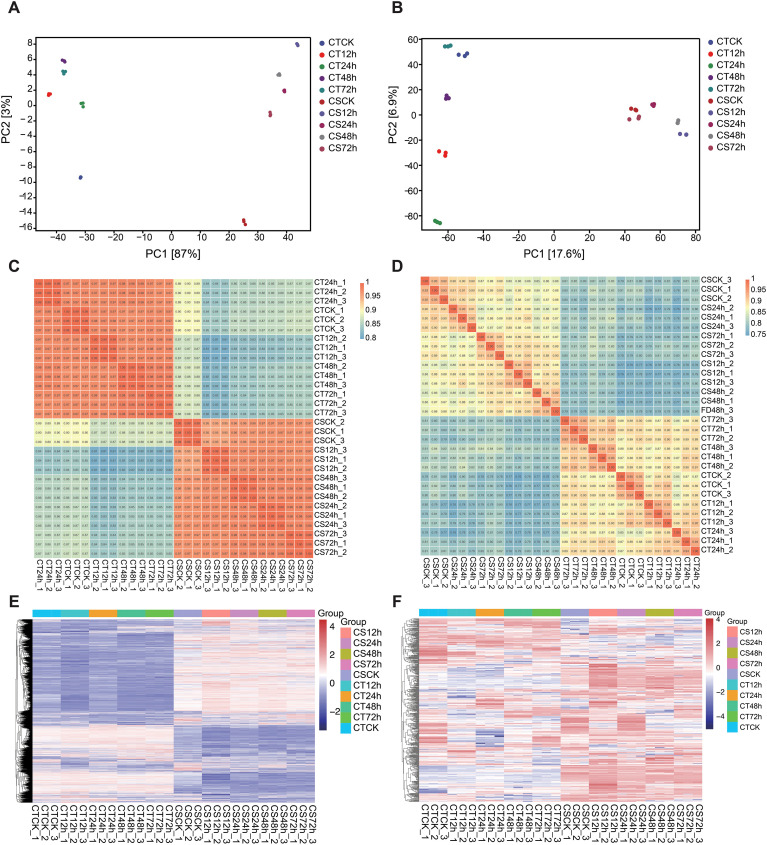
Global transcriptomic and metabolomic profiles of CT and CS under low-temperature stress. **(A)** Principal component analysis (PCA) of transcriptomic samples. **(B)** PCA of metabolomic samples. **(C)** Pearson’s correlation heatmap of transcriptomic samples. **(D)** Pearson’s correlation heatmap of metabolomic samples. **(E)** Hierarchical clustering heatmap of differentially expressed genes (DEGs). **(F)** Hierarchical clustering heatmap of differentially accumulated metabolites (DAMs). Samples were collected from CT and CS materials at 0 h (CK), 12 h, 24 h, 48 h, and 72 h under low-temperature treatment. Each treatment included three biological replicates.

To further clarify the molecular changes associated with the distinct low-temperature responses of CT and CS, transcriptomic and metabolomic sequencing was conducted at key time points. A total of 13,368 expressed genes (mean FPKM > 1) and 1,542 annotated metabolites were detected, including 7,842 differentially expressed genes (DEGs) and 553 differentially accumulated metabolites (DAMs). Detailed information is provided in **Supplementary Tables S6**, **S7**, respectively. PCA showed clear separation of transcriptomic and metabolomic samples across treatment stages and between CT and CS. Pearson’s correlation heatmaps further indicated high reproducibility among biological replicates within each group, while clustering analyses of DEGs and DAMs revealed distinct expression and accumulation patterns across time points and between the two material types (**Figure 3**). Together, these results indicate that low-temperature stress substantially reshaped the transcriptomic and metabolomic profiles of CT and CS, providing a reliable basis for subsequent comparative analyses. Sequencing quality-control results are provided in **Supplementary Tables S8**, **S9**.

**Figure 4:**
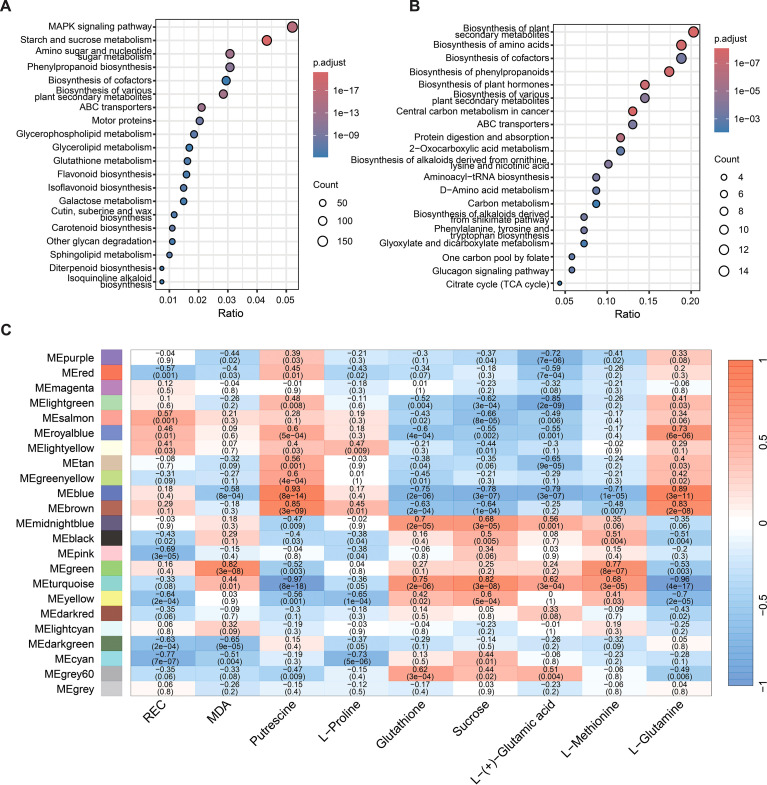
KEGG enrichment and WGCNA-based association analysis of CT and CS under low-temperature stress. **(A)** Kyoto Encyclopedia of Genes and Genomes (KEGG) enrichment analysis of differentially expressed genes (DEGs). **(B)** KEGG enrichment analysis of differentially accumulated metabolites (DAMs). **(C)** Correlation heatmap between weighted gene co-expression network analysis (WGCNA) modules and physiological traits or representative cold response-related metabolites. In panels **(A, B)**, dot size represents the number of DEGs or DAMs enriched in each pathway, and color indicates the adjusted P value. In panel **(C)**, values in each cell indicate correlation coefficients, and values in parentheses indicate P values. The color scale represents the strength and direction of the correlations. REC, relative electrolyte conductivity; MDA, malondialdehyde.

KEGG enrichment analysis showed that DEGs were mainly enriched in starch and sucrose metabolism, amino acid biosynthesis, phenylpropanoid biosynthesis, glutathione metabolism, ascorbate and aldarate metabolism, ATP-binding cassette (ABC) transporters, and the mitogen-activated protein kinase (MAPK) signaling pathway (**Figure 4A**). DAMs were mainly enriched in amino acid metabolism, sugar metabolism, glutathione- and ascorbate-related metabolism, biosynthesis of secondary metabolites, and carbon metabolism (**Figure 4B**). Notably, glutathione- and ascorbate-related pathways were enriched in both the transcriptomic and metabolomic datasets, suggesting that redox homeostasis is likely an important component of the low-temperature response. Detailed enrichment results are listed in **Supplementary Tables S10**, **S11**.

WGCNA was further performed using all expressed genes together with the physiological indices and cold tolerance-related metabolites described above. The turquoise, blue, and brown modules showed strong associations with cold adaptation (**Figure 4C**). These modules were significantly correlated with REC, MDA, and several representative metabolites, including putrescine, L-proline, glutathione, sucrose, L-(+)-glutamic acid, L-methionine, and L-glutamine, indicating that cold adaptation in *Sapindus mukorossi* may be closely associated with osmotic adjustment, antioxidant defense, and the reprogramming of sugar and amino acid metabolism. Detailed correlations between modules and physiological traits or metabolites are provided in **Supplementary Table S12**.

### 3.4 qRT-PCR validation of candidate genes and identification of *SmAPX2*

**Figure 5:**
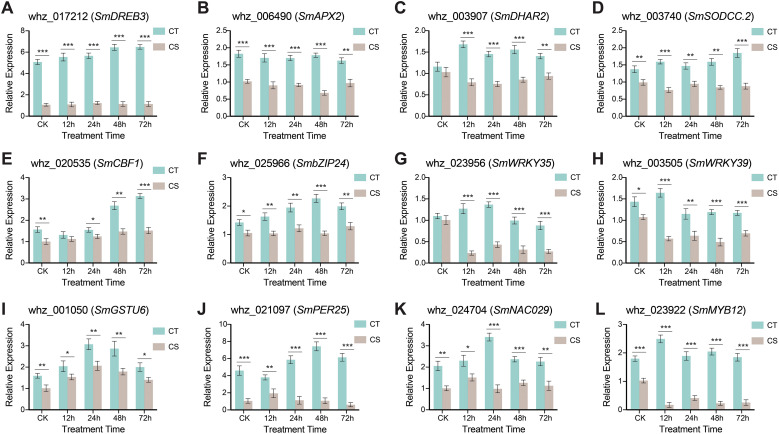
qRT-PCR validation of candidate cold-responsive genes in CT and CS under low-temperature stress. Relative expression levels of **(A)** *SmDREB3* (whz_017212), **(B)** *SmAPX2* (whz_006490), **(C)** *SmDHAR2* (whz_003907), **(D)** *SmSODCC.2* (whz_003740), **(E)** *SmCBF1* (whz_020535), **(F)** *SmbZIP24* (whz_025966), **(G)** *SmWRKY35* (whz_023956), **(H)** *SmWRKY39* (whz_003505), **(I)** *SmGSTU6* (whz_001050), **(J)** *SmPER25* (whz_021097), **(K)** *SmNAC029* (whz_024704), and **(L)** *SmMYB12* (whz_023922) in CT and CS after low-temperature treatment. Samples were collected at 0 (CK), 12, 24, 48, and 72 h. Data are presented as mean ± SD (n = 3). Asterisks indicate significant differences between CT and CS at the same time point according to Student’s t-test (*P < 0.05, **P < 0.01, ***P < 0.001).

Based on the cold adaptation-related modules identified by WGCNA, together with gene expression levels and functional annotation, 12 candidate cold-responsive genes were selected for qRT-PCR validation to compare their expression patterns between CT and CS (**Figure 5**). These included the transcription factor genes *SmDREB3*, *SmCBF1*, *SmbZIP24*, *SmWRKY35*, *SmWRKY39*, *SmNAC029*, and *SmMYB12*, as well as the functional genes *SmAPX2*, *SmDHAR2*, *SmSODCC*.2, *SmGSTU6*, and *SmPER25*. Detailed information on these candidate genes is provided in **Supplementary Table S13**. qRT-PCR analysis showed clear material-dependent differences in the expression of the 12 candidate genes during low-temperature treatment, with most genes exhibiting higher transcript levels in CT than in CS (**Figure 5**). Several transcription factor genes, including *SmDREB3*, *SmWRKY35*, *SmWRKY39*, *SmNAC029*, and *SmMYB12*, displayed sustained expression or marked induction in CT. A similar pattern was observed for antioxidant- and redox-related genes, such as *SmAPX2*, *SmDHAR2*, *SmSODCC.2*, *SmGSTU6*, and *SmPER25*, which showed higher transcript abundance in CT across most time points. Overall, most validated candidate genes showed stronger or more sustained induction in CT than in CS under low-temperature treatment. Among the candidate genes, *SmAPX2* showed a distinct expression pattern. Its transcript level remained relatively high throughout the treatment period and was consistently higher in CT than in CS. Together with the previous KEGG enrichment results from the transcriptomic and metabolomic analyses, which showed common enrichment of glutathione- and ascorbate-related pathways during the low-temperature response, these data further support the potential involvement of *SmAPX2* in ROS scavenging and the maintenance of redox homeostasis. Therefore, *SmAPX2* was selected as a key candidate gene for subsequent functional validation.

### 3.5 Functional validation of *SmAPX2*

Before functional validation, the putative subcellular localization of *SmAPX2* was predicted using four online tools. Three tools predicted *SmAPX2* to be cytoplasmic, whereas Plant-mPLoc suggested a possible peroxisomal localization (**Supplementary Table S14**). Therefore, *SmAPX2* was tentatively considered most likely a cytoplasm-localized APX-like protein in this study, but its precise subcellular localization remains to be experimentally confirmed.

**Figure 6:**
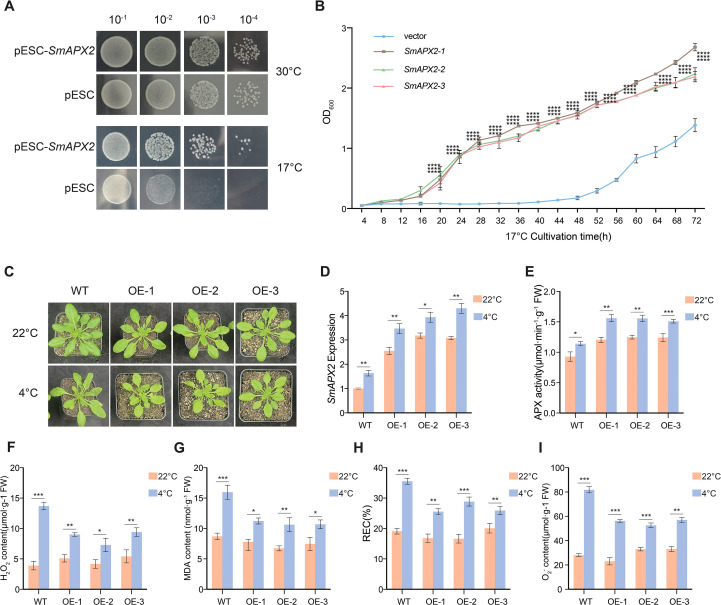
Functional validation of *SmAPX2* in yeast and transgenic *Arabidopsis thaliana*. **(A)** Spot assay of yeast cells carrying pESC-*SmAPX2* or the empty vector (pESC) under 30 and 17 °C conditions. Serial 10-fold dilutions are indicated above the panels. **(B)** Growth curves of the empty-vector control and three *SmAPX2*-expressing yeast lines cultured at 17 °C. **(C)** Phenotypes of wild-type (WT) and *SmAPX2*-overexpressing *Arabidopsis* lines (OE-1, OE-2, and OE-3) under 22 °C and after 4 °C treatment for 24 h. **(D)** Relative expression level of *SmAPX2*. **(E)** Ascorbate peroxidase (APX) activity. **(F)** Hydrogen peroxide (H_2_O_2_) content. **(G)** Malondialdehyde (MDA) content. **(H)** Relative electrolyte conductivity (REC). **(I)** Superoxide anion (O_2_·⁻) content. In panels **(D–I)**, orange bars represent 22 °C and blue bars represent 4 °C. Data are presented as mean ± SD (n = 3). Asterisks indicate significant differences between 22 °C and 4 °C within the same genotype according to Student’s t-test (*P < 0.05, **P < 0.01, ***P < 0.001). In **(B)**, asterisks indicate significant differences between the *SmAPX2*-expressing lines and the empty-vector control at the same time point according to Student’s t-test.

To evaluate the potential role of *SmAPX2* in low-temperature tolerance, heterologous expression analysis in yeast and overexpression assays in *Arabidopsis thaliana* were performed (**Figure 6**). In the yeast heterologous expression system, no obvious growth difference was observed between the pESC-*SmAPX2* recombinant strain and the empty-vector control under different dilution gradients at 30 °C. In contrast, under 17 °C conditions, the recombinant strain showed better growth than the empty-vector control, as indicated by better colony formation at higher dilution gradients (**Figure 6A**). Consistent with the spot assay, the liquid culture assay at 17 °C showed that the three *SmAPX2*-expressing yeast lines grew faster than the empty-vector control (**Figure 6B**). Their OD_600_ values increased rapidly after 16 h and remained higher than that of the control throughout the subsequent culture period. These results indicate that heterologous expression of SmAPX2 improved yeast growth under low-temperature conditions.

To further validate its function in planta, *SmAPX2* was overexpressed in Arabidopsis. Under normal conditions (22 °C), the three independent overexpression lines (OE-1, OE-2, and OE-3) showed no obvious phenotypic differences from the wild type (WT). After treatment at 4 °C for 24 h, however, the overexpression lines displayed better overall growth than WT, with less severe leaf wilting and chilling injury (**Figure 6C**). qRT-PCR analysis confirmed that *SmAPX2* was highly expressed in the overexpression lines and was further induced by low-temperature treatment (**Figure 6D**). In parallel, APX activity increased after 4 °C treatment in the overexpression lines and remained higher than that in WT (**Figure 6E**). Further measurements of oxidative damage- and membrane injury-related indices showed that H_2_O_2_ content, MDA content, REC, and O_2_·⁻ content increased after low-temperature treatment relative to the 22 °C control in both WT and the overexpression lines, but the increases were smaller in the overexpression lines than in WT (**Figures 6F–I**). Under 4 °C conditions, the overexpression lines accumulated lower levels of H_2_O_2_, MDA, REC, and O_2_·⁻ than WT, indicating less oxidative damage and membrane injury.

### 3.6 Transcriptional activation of *SmAPX2* by *SmWRKY4*

**Figure 7:**
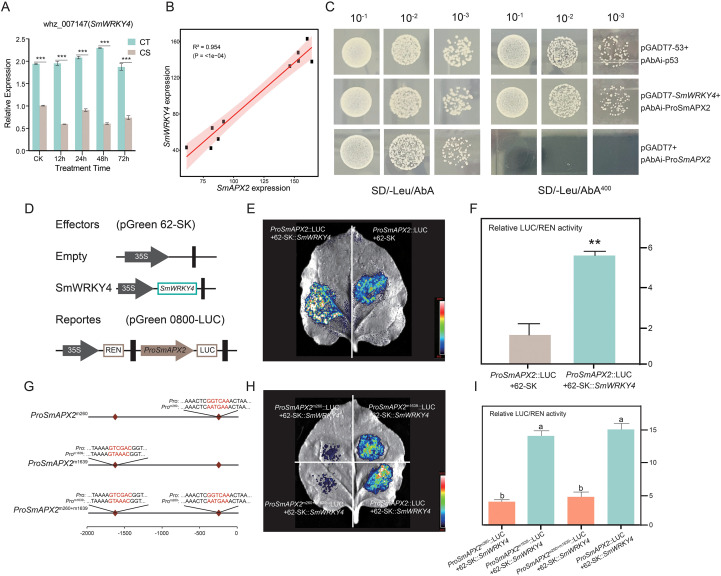
Transcriptional activation of the *SmAPX2* promoter by *SmWRKY4.* **(A)** Relative expression level of *SmWRKY4* (whz_007147) in CT and CS under low-temperature treatment. **(B)** Correlation analysis between *SmWRKY4* and *SmAPX2* expression levels. **(C)** Yeast one-hybrid assay showing the interaction between *SmWRKY4* and the *SmAPX2* promoter fragment under AbA selection. pGADT7-53 + pAbAi-p53 was used as the positive control, and pGADT7 + pAbAi-ProSmAPX2 was used as the negative control. **(D)** Schematic diagrams of the effector and reporter constructs used in the dual-luciferase assay. **(E)** Luminescence imaging of *Nicotiana benthamiana* leaves co-infiltrated with the indicated constructs. **(F)** Relative LUC/REN activity of the *SmAPX2* promoter activated by *SmWRKY4*. **(G)** Schematic diagram of W-box mutations in the *SmAPX2* promoter. The W-box core sequence TGAC was mutated to TAAC. **(H)** Luminescence imaging of *N. benthamiana* leaves co-infiltrated with *SmWRKY4* and wild-type or mutated *SmAPX2* promoter reporters. **(I)** Relative LUC/REN activity of wild-type and mutated *SmAPX2* promoters in the presence of *SmWRKY4*. Data are presented as mean ± SD (n = 3). Asterisks indicate significant differences between CT and CS at the same time point in panel **(A)**, or between the empty-vector control and *SmWRKY4* effector treatment in panel **(F)**, according to Student’s t-test (**P < 0.01, ***P < 0.001). Different lowercase letters in panel **(I)** indicate significant differences among promoter constructs according to one-way ANOVA followed by Duncan’s multiple range test at *P* < 0.05 **P < 0.01, ***P < 0.001.

To clarify the upstream transcriptional regulation of *SmAPX2*, cis-element analysis was performed on its promoter sequence. WRKY transcription factor-binding sites were identified in the *SmAPX2* promoter. Combined with transcriptome annotation and low-temperature-responsive expression patterns, *SmWRKY4* was further examined as a potential upstream regulator (**Figure 7**). qRT-PCR analysis showed that *SmWRKY4* expression was consistently higher in CT than in CS throughout the low-temperature treatment (**Figure 7A**). Expression levels of *SmWRKY4* and *SmAPX2* were closely correlated (R² = 0.954, *P* < 0.0001; **Figure 7B**), with both genes showing similar expression trends under low-temperature conditions. To examine the interaction between *SmWRKY4* and the *SmAPX2* promoter, a yeast one-hybrid (Y1H) assay was performed. Under the screening condition of 400 ng·mL⁻¹ Aureobasidin A (AbA), yeast cells co-transformed with *SmWRKY4* and Pro*SmAPX2* grew normally, whereas the negative control did not (**Figure 7C**), indicating that *SmWRKY4* could bind to the tested *SmAPX2* promoter fragment. The transcriptional regulatory activity of *SmWRKY4* was further examined using a dual-luciferase transient expression assay. Compared with the empty-vector control, co-expression of *SmWRKY4* and Pro*SmAPX2*::LUC significantly enhanced the luminescence signal in *Nicotiana benthamiana* leaves and increased relative luciferase activity (LUC/REN) (**Figures 7D–F**), indicating that *SmWRKY4* activated the *SmAPX2* promoter.

To further determine whether the predicted W-box elements contributed to *SmWRKY4*-mediated activation of *SmAPX2*, mutation analysis was performed using dual-luciferase assays. Two W-box elements located at approximately −260 bp and −1639 bp in the *SmAPX2* promoter were mutated individually or simultaneously (**Figure 7G**). Compared with the wild-type promoter, mutation of the proximal W-box at −260 bp significantly reduced *SmWRKY4*-induced LUC/REN activity, whereas mutation of the distal W-box at −1639 bp did not markedly affect promoter activation (**Figures 7H, I**). Simultaneous mutation of both W-box elements also reduced promoter activity to a level similar to that of the −260 bp mutation. These results suggest that the proximal W-box at −260 bp is the major cis-element contributing to *SmWRKY4*-mediated activation of the *SmAPX2* promoter.

## 4 Discussion

Cold adaptation in woody plants is shaped by coordinated changes at the structural, physiological, and molecular levels. In this study, CT and CS materials of *Sapindus mukorossi* were compared using anatomical observation, physiological and biochemical measurements, multi-omics analysis, functional assays, and regulatory validation. Across these datasets, CT differed from CS in branch integrity, cellular status, antioxidant behavior, metabolic adjustment, and gene regulation. These differences support the view that the stronger cold tolerance of CT is associated with better maintenance of branch structure, more stable redox balance during prolonged low-temperature exposure, and coordinated molecular regulation involving metabolic reprogramming and the *SmWRKY4*–*SmAPX2* module.

### 4.1 Structural stability and cellular protection underpin the superior cold tolerance of CT

In perennial woody plants, branches are directly exposed to winter chilling and freezing while also retaining the tissues required for subsequent regrowth. Their structural condition therefore has direct relevance to overwintering performance. Consistent with this view, CT showed visible bud break after natural overwintering, whereas no sprouted buds were observed in CS within the evaluated branch segment. In the present study, CT maintained clearer tissue stratification, tighter cellular arrangement, and better vascular organization than CS after natural low-temperature exposure. By contrast, CS showed more evident tissue loosening, local collapse, and phloem disorganization. This contrast indicates that the difference between CT and CS was already apparent at the level of branch tissue stability. Quantitative anatomical measurements further showed that CT had greater cortex and phloem thickness and higher cortex and phloem proportions, whereas branch radius, xylem thickness, and xylem rate did not differ significantly between CT and CS. This suggests that the anatomical advantage of CT was mainly associated with better preservation of cortical and phloem tissues rather than with overall branch size or xylem development. The structural differences were accompanied by corresponding physiological changes. Compared with CS, CT maintained lower REC and MDA levels and accumulated higher amounts of Pro, SS, and SP, indicating better membrane stability and stronger osmotic protection. Similar patterns have been reported in other species, where proline and soluble sugars contribute to osmotic adjustment as well as membrane stabilization under low-temperature conditions, while soluble proteins may reflect the accumulation of protective components (Wanner and Junttila, 1999; Gu et al., 2021; Zhang et al., 2022). In this context, the advantage of CT is better understood as a more stable structural and cellular state than as a difference in any single physiological index.

### 4.2 Superior cold tolerance in CT is associated with more sustained ROS buffering

Disruption of ROS homeostasis is a central feature of cold-induced cellular injury (Gechev and Petrov, 2020). Under low-temperature conditions, reduced membrane fluidity, altered electron transport, and metabolic imbalance can promote ROS accumulation, which then accelerates membrane lipid peroxidation and aggravates cellular damage. Against this background, the key difference between CT and CS was not simply whether an antioxidant response occurred, but whether that response could be sustained as stress duration increased (Gu et al., 2021; Feng et al., 2025). This distinction was evident in antioxidant enzyme dynamics. SOD and POD activities were generally higher in CT during the middle and late stages of treatment, whereas APX remained at a higher level in CT from 12 h onward. CAT showed a more variable pattern, with a transiently higher value in CS at 8 h but higher activity in CT at 12 and 24 h. These changes suggest that CS was able to initiate an early antioxidant response, but that this response weakened as stress persisted. CT, by contrast, maintained a more sustained antioxidant profile, especially during the later phase of treatment. When considered together with lower REC and MDA and higher Pro, SS, and SP in CT, this pattern suggests that the physiological advantage of CT lies more in response persistence than in short-term response intensity (Zhang et al., 2022).

### 4.3 Cold adaptation in *Sapindus mukorossi* involves coordinated transcriptional and metabolic reprogramming

Phenotypic and physiological analyses clearly separated CT from CS, but these observations alone could not explain how this divergence was established at the molecular level. The transcriptomic and metabolomic data extended these observations by showing clear separation between CT and CS in PCA, correlation analysis, and clustering patterns, indicating that the difference in cold response was accompanied by substantial transcriptional and metabolic divergence (Liu et al., 2025). Joint enrichment of DEGs and DAMs in starch and sucrose metabolism, amino acid biosynthesis, phenylpropanoid biosynthesis, MAPK signaling, and AsA–GSH-related pathways points to coordinated metabolic and regulatory adjustment rather than isolated activation of a few stress genes. Sugar metabolism in this context is relevant not only to osmotic adjustment but also to energy balance under prolonged stress, whereas amino acid metabolism may contribute to nitrogen redistribution, osmoprotection, and redox adjustment (Luo et al., 2025). Among the enriched pathways, AsA–GSH-related processes are especially important because they connect directly with the physiological results. WGCNA further linked key modules with REC, MDA, Pro, sucrose, glutathione, and several amino acid-related metabolites, suggesting that cold adaptation in *S. mukorossi* depends on coordinated transcriptional and metabolic reorganization rather than on a single detoxification process (Langfelder and Horvath, 2008; Lu et al., 2023; Luo et al., 2025).

### 4.4 *SmAPX2* functions as a key effector linking antioxidant defense to cold tolerance

Among the candidate genes identified in this study, *SmAPX2* stood out because several lines of evidence converged on it. Its expression remained higher in CT than in CS during low-temperature treatment, its annotated function matched the commonly enriched AsA–GSH pathway, and its role was further supported by heterologous expression and overexpression analyses. *SmAPX2* is therefore better interpreted as a functionally relevant effector than as a candidate identified solely by omics screening. The validation results were internally consistent. In yeast, heterologous expression of *SmAPX2* improved growth under low-temperature conditions. In *Arabidopsis*, overexpression of *SmAPX2* increased APX activity and reduced the accumulation of H_2_O_2_, MDA, REC, and O_2_·⁻ after cold treatment. These data indicate that *SmAPX2* contributes to low-temperature tolerance by strengthening ROS detoxification and reducing membrane injury. Given the known role of APX in H_2_O_2_ removal within the AsA–GSH cycle, this interpretation is also consistent with the physiological and multi-omics evidence obtained here (Zeng et al., 2022; Li et al., 2023; Foyer and Kunert, 2024; Yoshimura and Ishikawa, 2024). At the same time, the current functional evidence comes mainly from heterologous systems, and the quantitative contribution of *SmAPX2* in the native background of *S. mukorossi* remains unresolved.

The predicted subcellular localization provides additional context for interpreting the function of *SmAPX2*. Most prediction tools suggested that *SmAPX2* is cytoplasmic, although Plant-mPLoc predicted a possible peroxisomal localization. This result indicates that *SmAPX2* may mainly function in the cytoplasm, but it does not exclude possible localization in other ROS-related compartments. Cytosolic APX isoforms are important components of the cellular H_2_O_2_-scavenging system and contribute to the maintenance of redox homeostasis in the cytosolic compartment. In this study, *SmAPX2* overexpression increased APX activity and reduced the accumulation of H_2_O_2_, O_2_·⁻, MDA, and REC under low-temperature stress, which is consistent with a role in ROS detoxification. However, because the prediction results were not fully consistent and GFP-based localization was not performed, the precise localization of *SmAPX2* remains to be verified.

### 4.5 The *SmWRKY4–SmAPX2* module is associated with cold adaptation in *Sapindus mukorossi*

If *SmAPX2* acts as a downstream effector, *SmWRKY4* appears to represent part of the upstream regulatory layer. Multiple WRKY-binding sites were identified in the *SmAPX2* promoter, and this prediction was supported by expression and validation data (Jiang et al., 2017). *SmWRKY4* showed consistently higher expression in CT than in CS, was strongly correlated with *SmAPX2*, bound to the *SmAPX2* promoter fragment in the Y1H assay, and activated promoter activity in the dual-luciferase assay. These results indicate that the proposed *SmWRKY4*–*SmAPX2* module is more than a simple co-expression relationship and suggest that the difference between CT and CS involves not only downstream defense capacity but also upstream transcriptional activation (Wang et al., 2022, Wang et al., 2025b). Even so, the current model remains provisional because the broader regulatory range of *SmWRKY4* and the *in vivo* contribution of this regulatory relationship remain unresolved.

The W-box mutation analysis further refined this regulatory relationship. Among the two predicted W-box elements tested, mutation of the proximal W-box at −260 bp markedly weakened *SmWRKY4*-mediated promoter activation, whereas mutation of the distal W-box at −1639 bp had little effect. This result suggests that not all predicted W-box motifs in the *SmAPX2* promoter contribute equally to transcriptional activation by *SmWRKY4*, and that the proximal W-box may serve as the major functional site. Such site-specific effects are consistent with the view that transcription factor binding and activation depend not only on motif presence, but also on motif position, surrounding sequence context, and promoter architecture. Nevertheless, because the W-box mutation assay was performed in a transient dual-luciferase system, further experiments such as EMSA, ChIP-qPCR, or loss-of-function analysis in *S. mukorossi* would be needed to further verify binding specificity, *in vivo* promoter occupancy, and the functional contribution of this regulatory module.

### 4.6 Conserved and woody plant-specific features of cold adaptation in *Sapindus mukorossi*

Compared with model plants such as *Arabidopsis thaliana*, cold adaptation in *S. mukorossi* shares core features such as transcriptional reprogramming, osmotic adjustment, and antioxidant activation. In Arabidopsis, these processes involve coordinated regulation of freezing tolerance, the CBF-centered cold-response network, and calcium-mediated signaling (Thomashow, 1999, Thomashow, 2010; Peng et al., 2024). In this context, the *SmWRKY4*–*SmAPX2* relationship identified here is better understood as part of a conserved link between transcriptional regulation, ROS detoxification, and cold adaptation. At the same time, as a perennial woody species, *S. mukorossi* appears to rely more strongly on branch structural integrity, tissue preservation, and sustained buffering during prolonged low-temperature exposure and overwintering. In woody plants, frost resistance depends not only on cellular adjustment but also on organ- and tissue-level stability, which directly affects winter survival and recovery (Neuner, 2014). Thus, the cold adaptation mechanism of *S. mukorossi* appears to combine conserved cellular stress responses with woody plant-specific requirements for branch and vascular tissue maintenance.

## 5 Conclusion

This study shows that cold adaptation in *Sapindus mukorossi* is a coordinated process involving structural maintenance, osmotic adjustment, antioxidant defense, and molecular regulation. Compared with CS, CT maintained greater branch structural stability, stronger cellular protection, and a more sustained capacity for reactive oxygen species (ROS) scavenging and redox homeostasis under low-temperature stress. Multi-omics analyses further indicated that these differences were closely associated with coordinated reprogramming of sugar and amino acid metabolism, together with remodeling of glutathione- and ascorbate-related pathways. Functional validation showed that *SmAPX2* enhanced low-temperature tolerance by increasing APX activity and reducing the accumulation of H_2_O_2_, MDA, REC, and O_2_·⁻. Regulatory analyses showed that *SmWRKY4* bound to the *SmAPX2* promoter and activated its transcriptional activity, with the proximal W-box contributing substantially to *SmWRKY4*-mediated promoter activation. Taken together, these findings support the involvement of the *SmWRKY4*–*SmAPX2* module in cold adaptation-associated antioxidant responses in *S. mukorossi* and provide candidate targets for cold-tolerant germplasm evaluation and future molecular improvement. However, loss-of-function validation in the native *S. mukorossi* background will be required to further determine the specific contribution of this module to cold tolerance.

## Data Availability

The datasets presented in this study can be found in online repositories. The names of the repository/repositories and accession number(s) can be found below: https://www.ncbi.nlm.nih.gov/search/all/?term=PRJNA1438190, PRJNA1438190.
